# Population-based coverage survey results following the mass drug administration of azithromycin for the treatment of trachoma in Amhara, Ethiopia

**DOI:** 10.1371/journal.pntd.0006270

**Published:** 2018-02-16

**Authors:** Tigist Astale, Eshetu Sata, Mulat Zerihun, Andrew W. Nute, Aisha E. P. Stewart, Demelash Gessese, Gedefaw Ayenew, Berhanu Melak, Melsew Chanyalew, Zerihun Tadesse, E. Kelly Callahan, Scott D. Nash

**Affiliations:** 1 Trachoma Control Program, The Carter Center, Addis Ababa, Ethiopia; 2 Trachoma Control Program, The Carter Center, Atlanta, Georgia, United States of America; 3 Health Promotion and Disease Prevention Core Process, The Amhara Regional Health Bureau, Bahir Dar, Ethiopia; University of California San Francisco, UNITED STATES

## Abstract

**Background:**

Trachoma is the leading infectious cause of blindness worldwide. In communities where the district level prevalence of trachomatous inflammation-follicular among children ages 1–9 years is ≥5%, WHO recommends annual mass drug administration (MDA) of antibiotics with the aim of at least 80% coverage. Population-based post-MDA coverage surveys are essential to understand the effectiveness of MDA programs, yet published reports from trachoma programs are rare.

**Methods:**

In the Amhara region of Ethiopia, a population-based MDA coverage survey was conducted 3 weeks following the 2016 MDA to estimate the zonal prevalence of self-reported drug coverage in all 10 administrative zones. Survey households were selected using a multi-stage cluster random sampling design and all individuals in selected households were presented with a drug sample and asked about taking the drug during the campaign. Zonal estimates were weighted and confidence intervals were calculated using survey procedures. Self-reported drug coverage was then compared with regional reported administrative coverage.

**Results:**

Region-wide, 24,248 individuals were enumerated, of which, 20,942 (86.4%) individuals were present. The regional self-reported antibiotic coverage was 76.8% (95%Confidence Interval (CI):69.3–82.9%) in the population overall and 77.4% (95%CI = 65.7–85.9%) among children ages 1–9 years old. Zonal coverage ranged from 67.8% to 90.2%. Five out of 10 zones achieved a coverage >80%. In all zones, the reported administrative coverage was greater than 90% and was considerably higher than self-reported MDA coverage. Main reasons reported for MDA campaign non-attendance included being physically unable to get to MDA site (22.5%), traveling (20.6%), and not knowing about the campaign (21.0%). MDA refusal was low (2.8%) in this population.

**Conclusions:**

Although self-reported MDA coverage in Amhara was greater than 80% in some zones, programmatic improvements are warranted throughout Amhara to achieve higher coverage. These results will be used to enhance community mobilization and improve training for MDA distributors and supervisors to improve coverage in future MDAs.

## Author summary

Mass drug administration (MDA) with antibiotics is a key component of the trachoma control strategy. The World Health Organization (WHO) recommends that at least 80% of the target population should be reached with MDA. Drug coverage estimates from population-based surveys may increase our understanding of factors affecting the effectiveness of MDA. We conducted a region-wide population-based survey to estimate the prevalence of self-reported drug coverage in all ten administrative zones of Amhara region, an area with a population of approximately 21 million people. The self-reported drug coverage was greater than 80% in five of the ten zones and was 76.8% region-wide. Zonal administrative coverage reports were greater than 90% and were considerably higher than self-reported coverage in all zones. The discrepancy between administrative and self-report coverages also suggest that efforts should be made to better understand the reasons for the disparity in the two measures. The main reasons reported for not attending the MDA included being physically unable to get to the distribution site, traveling during the campaign, and lack of knowledge about the campaign. These findings suggest that making the distribution site accessible to all individuals, informing constituents about timing of the campaign to allow for travel, and providing adequate information about the campaign would improve MDA participation.

## Introduction

Trachoma, caused by ocular infection with *Chlamydia trachomatis*, is the leading infectious cause of blindness, estimated to be responsible for approximately 3% of all blindness worldwide [1]. The World Health Organization (WHO) estimates that 190.2 million people live in trachoma-endemic areas [2].

WHO recommends the SAFE strategy for trachoma control: Surgery for trachomatous trichiasis, Antibiotic distribution to treat *Chlamydia trachomatis* infection, and Facial cleanliness and Environmental improvement to reduce transmission of *Chlamydia trachomatis* [3]. Antibiotic distribution involves annual mass drug administration (MDA) with oral azithromycin or topical tetracycline eye ointment (TEO) to all communities within districts where the prevalence of trachomatous inflammation-follicular (TF) among children ages 1–9 years is ≥5% [3–5]. Furthermore, WHO recommends that programs reach at least 80% coverage during trachoma MDA campaigns [4].

Typically, trachoma programs monitor antibiotic coverage through use of routine administrative reports, whereby coverage is calculated as the number of doses administered, often recorded in registration books, divided by the total population often calculated using census records [6,7]. However, administrative data may be inaccurate due to incomplete registration books, lost or misused drugs being counted as consumed, or old, inaccurate, or missing census data. Some neglected tropical disease (NTD) programs have begun to use cluster random sample surveys to assess drug coverage through self-reporting [6–10]. These survey methods allow for population-based estimates that are not reliant on population data, which may be unreliable in many settings. Recently, the WHO Strategic and Technical Advisory Group for NTDs recommended the use of coverage survey evaluations based on self-report to estimate preventive chemotherapy coverage [11].

The Trachoma Control Program in Amhara National Regional State, Ethiopia, has been implementing the SAFE strategy throughout the entire region of Amhara since 2007 [12]. This region-wide population-based survey aimed to estimate the prevalence of self-reported drug coverage during the 2016 trachoma MDA, to compare self-reported coverage with reported administrative coverage, and to detail the characteristics of non-participation throughout Amhara, an area with a mature trachoma program serving approximately 21 million people. The results from this study can be used to improve MDA distribution and training throughout Amhara and will be instructive for other MDA programs.

## Methods

### Ethics statement

The study was approved by the ethical review committee of Amhara National Regional State Health Bureau, Ethiopia, and the Emory University Internal Review Board (IRB 079–2006). Permission was obtained from Woreda (district) Health Offices, kebele (sub district) representatives and gott (village) leaders. Verbal informed consent was obtained from the heads of households for household interviews and each individual and parents of minors were asked for consent/assent before questions were asked. Consent was recorded on electronic data collection tablets. All participant data was anonymized before data analysis.

### Mass drug administration

A MDA for trachoma was conducted in West Amhara sub-region (73 districts) in January 2016 and in East Amhara sub-region (70 districts) in May 2016. Only trachoma medications were administered during these MDAs. Including the 2016 distribution, all districts in Amhara have received six to eleven rounds of MDA. For each MDA, training, supervision and logistics are orchestrated by zonal health departments in each of the ten zones in Amhara, and administrative coverage is most often reported at that level. Although district level data is used to get approval for MDA treatment, conducting separate surveys for 167 districts of Amhara is logistically challenging. For these reasons, this survey was designed to estimate zonal level coverage to allow the program to appropriately target zones for future improvements.

The Amhara Regional Health Bureau conducted a zonal-level training of trainers before each MDA. Trainers for MDA were typically zonal and district health officers. Trainers then trained, or provided refresher training, to health extension workers (HEWs) and kebele leaders on MDA procedures before the start of the campaign. HEWs are village-based governmental health workers who typically live in the areas they serve. In general, there are two HEWs per kebele (population approximately 5,000 persons). Registration books were distributed to all villages by the district health offices, and village-specific census data, which are updated approximately every 5 years, were recorded in the books. The federal government was responsible for delivering MDA medications to districts, where they were then delivered to distribution points by district health offices. Village-based MDA distribution took place primarily at a central location, with occasional door-to-door distribution, following community mobilization by HEWs and volunteers. HEWs directly-observed treatment and recorded the doses distributed in the registration book. All consenting community members were offered either a single oral dose of azithromycin or a course of TEO according to national guidelines. TEO is provided for self-reporting pregnant women in their first trimester, children younger than six months, persons with serious illness, those who previously experienced adverse drug reaction to azithromycin and to participants who decline oral azithromycin. At the kebele level, HEWs adjusted the previous year’s population by subtracting deaths and emigration and by adding newly registered persons. Coverage was then aggregated to zonal levels.

### Study site and study period

The coverage survey was conducted in two phases: in February 2016, we surveyed 5 zones of West Amhara (estimated population of 13 million people); and in June 2016, we surveyed 5 zones of East Amhara (estimated population of 8.1 million people). This survey was conducted within three weeks of the 2016 MDA campaign.

### Study design

All districts that received MDA in 2016 were eligible for the coverage survey sampling procedure to produce zonal prevalence estimates of participation. Assuming a coverage estimate of 70% ± 5%, an alpha 0.05 and a design effect of 5.0, in each zone 1,615 participants were required to be surveyed. Accounting for non-response, the sample size was inflated by a factor of 1.15 to 1,857 participants. According to extensive trachoma impact surveys conducted in Amhara, there are on average 4.1 residents per household in Amhara, and therefore, 453 households per zone were required.

**Fig 1:**
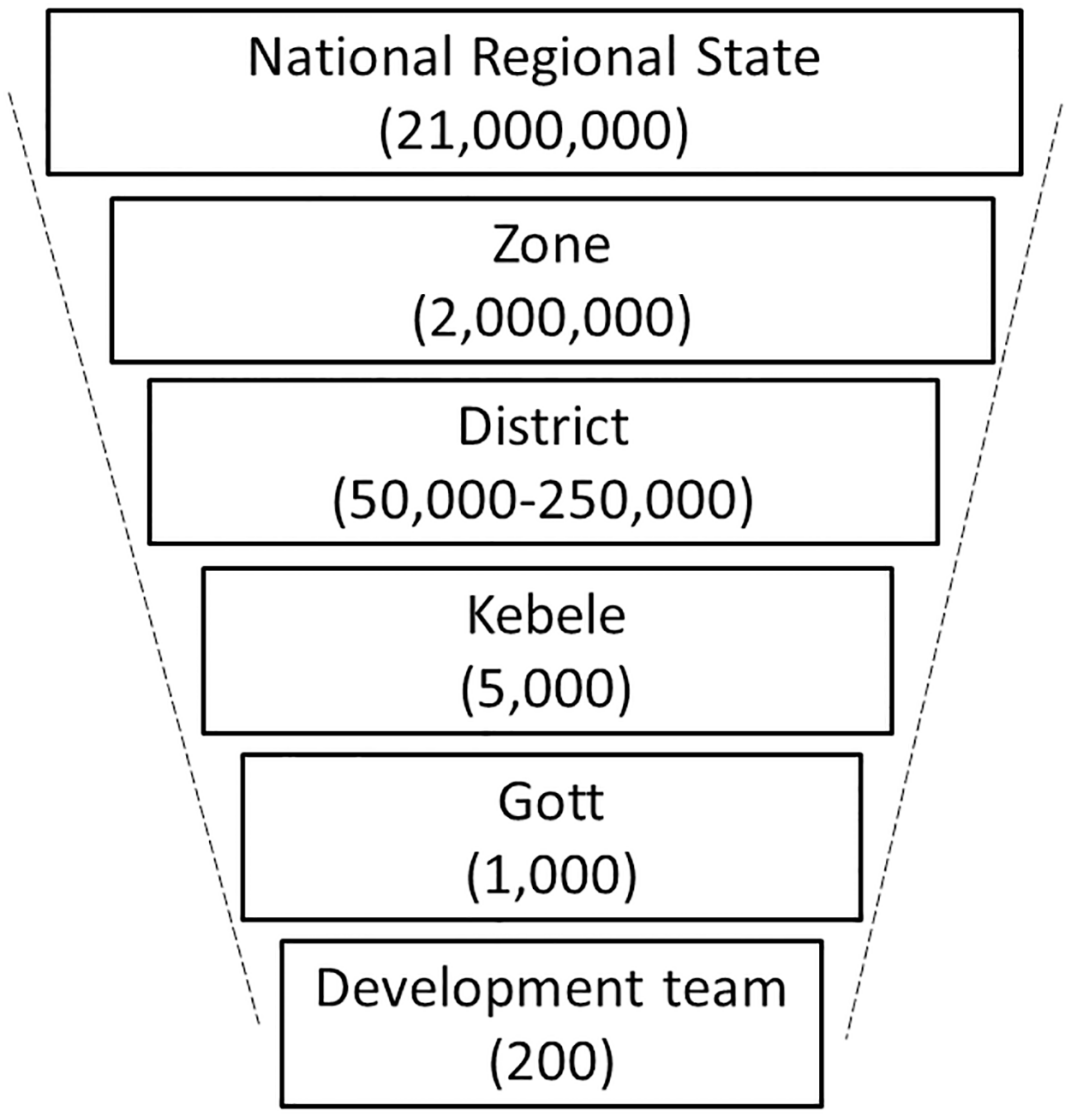
Administrative levels and generalized population of Amhara, Ethiopia, 2016.

To obtain the required number of households and participants, 32 clusters were selected in each zone, and 15 households were surveyed in each cluster using a multi-stage cluster random sampling design. In the first stage, four districts were randomly selected within each zone using probability proportional to estimated size (Fig 1). Next, four kebeles were selected within each district using probability proportional to estimated size; and then two gotts were randomly selected in each kebele. Gotts were further divided into administrative units called development teams (approximately 30 households). Each development team number was written on a folded piece of paper, and the gott leader blindly selected one. Each selected development team was then segmented into two segments of 15 households (roughly in half) using a sketch map technique; one segment was chosen randomly by the village representative; and all households in that segment were surveyed. When no one from a household was home during the time of the survey, the household was not replaced.

### Data collection

Data recorders participated in a 5-day training including a pilot study in the field. Data collection took place on Samsung Galaxy tablets running custom built software [13]. The household-level questionnaire assessed characteristics of the head of household including their self-reported use of azithromycin or TEO. In the census questionnaire, each household member was enumerated, shown azithromycin to enhance recall, and then asked if they were offered and took the medication during the campaign. Heads of household provided responses for young children. Neither HEWs nor MDA registration books were present at the time of household interviews. For individuals who were absent during the survey, a proxy question was asked to the head of the household or a respondent aged 18 years and older. Survey respondents who reported non-participation in the MDA were asked a series of questions to understand their non-participation.

### Statistical analyses

The primary outcome of the survey was the proportion of individuals who reported that they were offered and took azithromycin or TEO during the MDA campaign out of those present during the survey. The proxy responses for individuals who were not present during the survey were calculated and reported as a secondary outcome. Sampling weights were calculated based on the inverse of the probability of selection at each stage of selection. Zonal estimates were weighted, confidence intervals were calculated using Taylor linearization, and survey procedures (svy package) in Stata, version 13.1 (STATA Corporation, College Station TX, USA), were used to account for clustering. All percentages with confidence intervals were weighted estimates. Statistical comparisons were conducted using t-tests for continuous variables, and chi^2^ tests for categorical variables and accounted for the complex survey design when comparing weighted estimates. Maps were created in ArcGIS 10.4.1 (ESRI, Redlands, CA). Reported administrative coverage was provided by the Amhara Regional Trachoma Control Program.

## Results

### Survey responses

A total of 24,248 individuals from 5,184 households were enumerated from the 10 zones of Amhara. Among the enumerated individuals, 20,942 (86.4%) were present at the time of the survey, and 99.9% (20,932) of those present responded to the survey questions. The mean age of surveyed respondents was 23.8 years and 52.0% of the respondents were female. Individuals absent at the time of the survey were younger (mean age = 22.0 years, t = -4.909, Degrees of freedom (Df) = 24,220, P < 0.001) and more likely to be male, 67.8% (chi^2^ = 443.67, P < 0.001). The response rate was the highest (97.0%) in the age group less than 1 year, and lowest (80.6%) in the age group 10–19 years.

### Drug coverage

**Fig 2:**
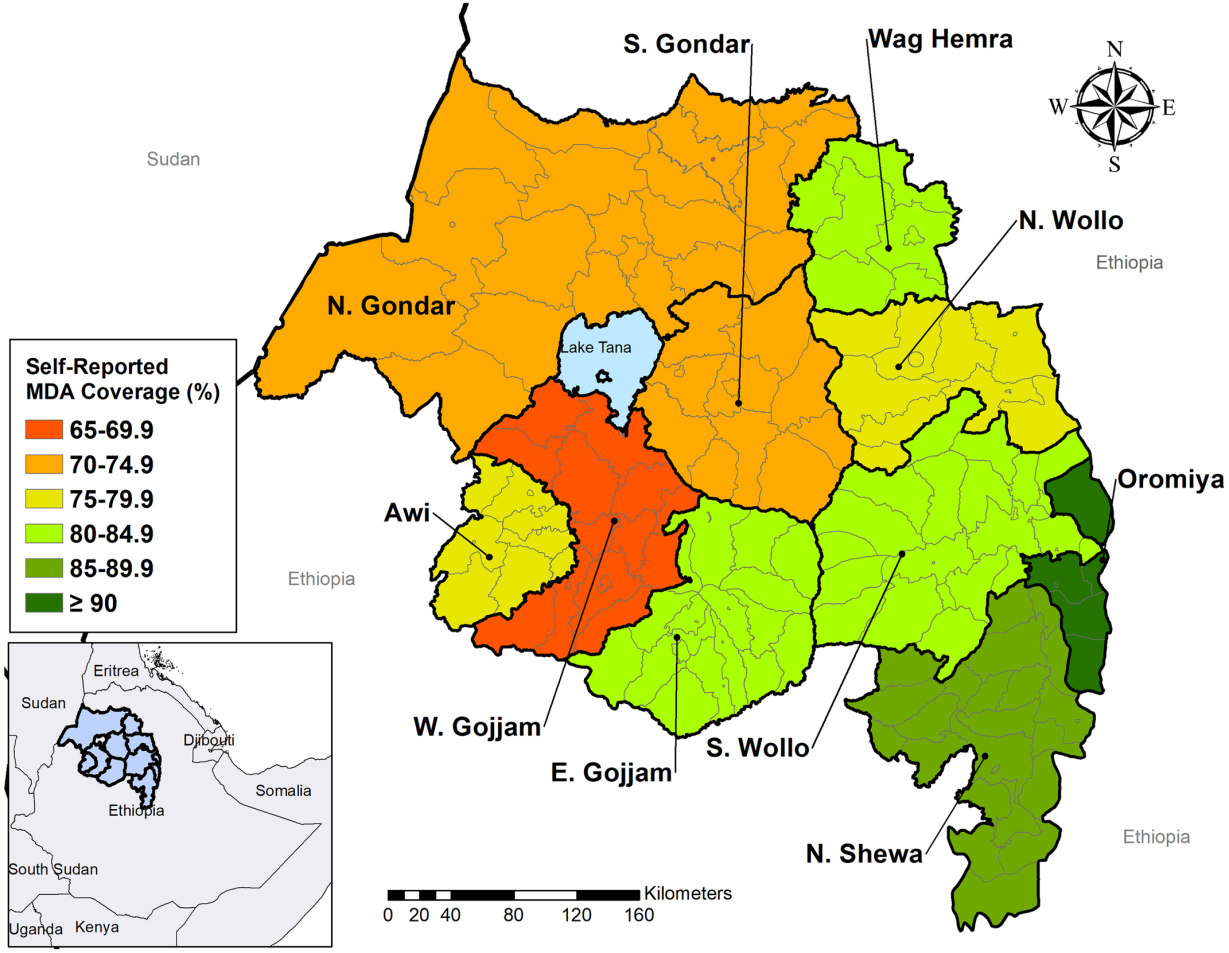
Geographic distribution of self-reported drug coverage by zone, Amhara, Ethiopia, 2016. Map created in ArcGIS 10.4.1 (ESRI, Redlands, CA) using shapefile sourced from the GADM database (gadm.org).

The regional self-reported drug coverage for individuals of all ages was 76.8% (95% Confidence Interval (CI): 69.3–82.9%) and ranged from 67.8% (95%CI: 55.7–77.8%) in West Gojjam zone to 90.2% (95%CI: 85.7–93.4%) in Oromia zone (Fig 2). Five out of ten zones had drug coverage greater than 80% among all age groups and among children ages 1–9 years (Table 1). No statistically significant differences in self-reported drug coverage were observed between males and females (77.8% vs. 75.9%; P = 0.14). The highest coverage was obtained among participants between 10–19 years, 81.1% (95% CI = 75.0–85.9%), while the coverage among children ages one to nine years was 77.4% (95%CI = 65.7–85.9%).

**Table 1:** Prevalence of self-reported drug coverage by zone, Amhara, Ethiopia, 2016.

Zone	Total N	Total N Households	Took Drug, % (95%CI) †	Took Drug, Age 1–9 years, (95%CI) †
Awi	1946	511	77.8% (64.5–87.1%)	78.9% (64.4–88.5%)
East Gojjam	2004	528	81.5% (67.0–90.5%)	86.0% (71.9–93.7%)
North Gondar	2139	499	70.5% (47.9–86.2%)	70.3% (47.6–86.1%)
South Gondar	2131	516	73.9% (66.3–80.3%)	78.0% (72.3–82.8%)
West Gojjam	2099	520	67.8% (55.7–77.8%)	72.6% (56.4–84.5%)
North Shoa	2125	524	85.1% (75.1–91.6%)	89.9% (85.0–93.4%)
North Wollo	2066	542	76.6% (43.0–93.4%)	68.1% (27.2–92.4%)
Oromia	2207	505	90.2% (85.7–93.4%)	92.6% (87.3–95.8%)
South Wollo	2091	534	81.9% (66.0–91.3%)	88.8% (70.9–96.3%)
Waghimra	2124	505	82.1% (71.5–89.4%)	84.8% (72.1–92.4%)

^†^ Weighted zonal estimate. Multilevel survey design accounted for in analysis

**Fig 3:**
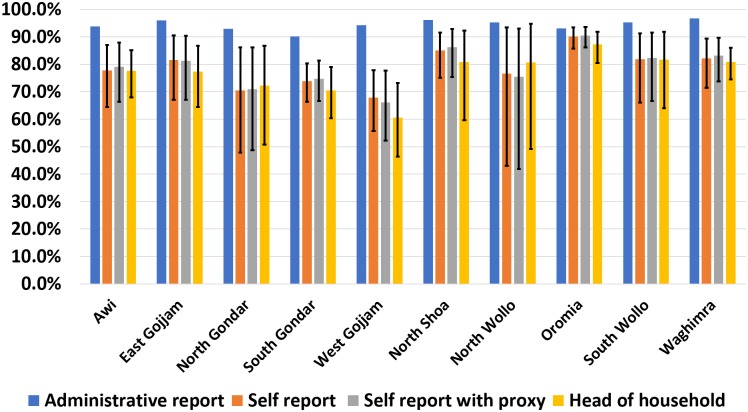
Administrative coverage, coverage estimates by self-report, by self-report with proxy responses, and by self-report of head of household by zone, Amhara, Ethiopia, 2016.

For individuals who were absent during the survey (n = 3,306), a proxy response (n = 2,784; 84%) was obtained from a respondent in the household. Regional drug coverage among those with a proxy response was 77.5% (95%CI: 67.9–84.8%). Calculating MDA coverage by combining both proxy and self-reported data, resulted in coverage similar to that calculated from self-reporting alone, with regional drug coverage of 76.9% (95%CI: 69.4–83.0%) (Fig 3). In the 2016 MDA campaign, the reported administrative coverage for Amhara region was higher than self-reported drug coverage and was greater than 90% in all ten zones. The discrepancy between the two estimates was the smallest in Oromia zone (2.9%) and greatest in West Gojjam (26.4%) zone. Head of household self-reported coverage was 75.8% (95%CI: 68.3–82.0%) for Amhara region and was similar to self-reported coverage among all individuals for all zonal estimates.

### Reasons for not taking drugs

Among participants present during the survey, 22.8% (95%CI: 16.6–30.3%) reported that they were not offered the drug. Among individuals who reported not being offered the drug, the most common reason given for not being offered was not attending the MDA campaign, 76.5% (95%CI: 63.7–85.8%) (Table 2). Regionally, the reasons reported for not attending the MDA campaign included being physically unable to get to the MDA site, 22.5% (95%CI: 15.0–32.3%), not knowing about the campaign, 21.0% (95%CI: 15.3–28.1%), and traveling during the campaign, 20.6% (95%CI: 15.5–27.0%). The most commonly reported reasons for not attending the MDA differed by zone (S1 Table). Among those who were offered the drug, only 0.6% (95%CI: 0.3–0.9%) (n = 64) reported not taking the drug.

**Table 2:** Reasons for not being offered drug, not attending MDA, and MDA refusal, Amhara, Ethiopia, 2016.

Characteristics	Percent (95%CI) †
Drug not offered	22.8% (16.6–30.3%)
Reasons for not being offered drug	
Did not attend the MDA campaign event	76.5% (63.7–85.8%)
Campaign did not come to my village	20.6% (11.5–34.1%)
Other	2.9% (1.8–4.8%)
Reasons for not attending MDA	
Physically unable to get to the distribution site	22.5% (15.0–32.3%)
Did not know about the MDA campaign	21.0% (15.3–28.1%)
Traveling during the MDA	20.6% (15.5–27.0%)
Did not want the medication	13.0% (8.9–18.6%)
Chores or duties for the household	11.6% (9.3–14.5%)
Attended social or religious events elsewhere	10.1% (7.2–13.9%)
Other	1.2% (0.5–2.7%)
Drug offered but not taken	0.6% (0.3–0.9%)
Refused MDA*	2.8% (2.3–3.4%)
Reasons for refusal	
Has a serious illness/allergy	41.2% (31.9–51.1%)
Think I am not sick	3.5% (1.9–6.5%)
Side effect during previous campaign	3.2% (1.6–6.4%)
Do not think I will get the disease	2.4% (1.2–4.6%)
Lack of information about the medicine	1.9% (0.8–4.5%)
Tired of taking the pills	0.3% (0.1–1.0%)
Other	47.6% (38.3–57.0%)

^†^ Weighted zonal estimate. Multilevel survey design accounted for in analysis

*Includes those who reported not wanting the medication when asked for their reason for not attending the MDA campaign event, as well as those who reported that they were offered the medication but did not take it.

The regional prevalence of MDA refusal (people who did not attend the campaign because they did not want to receive the drug (n = 459) or who did not take the drug once it was offered (n = 64)) was 2.8% (95%CI: 2.3–3.4%), zone range: 1.1%-4.7%. The most commonly reported reasons for refusing were "Other", 47.6% (95%CI: 38.3–57.0%), and serious illness/allergy, 41.2% (95%CI: 31.9–51.1%). In anecdotal discussions with data recorders, the most common responses they classified as "Other" were related to various types of side effects experienced during previous MDAs. Females were more likely to refuse MDA than males (3.9% vs. 1.7%; P<0.0001), and the highest prevalence of refusal was in children less than 1 year old (6.6%, 95%CI: 4.1–10.4%).

**Fig 4:**
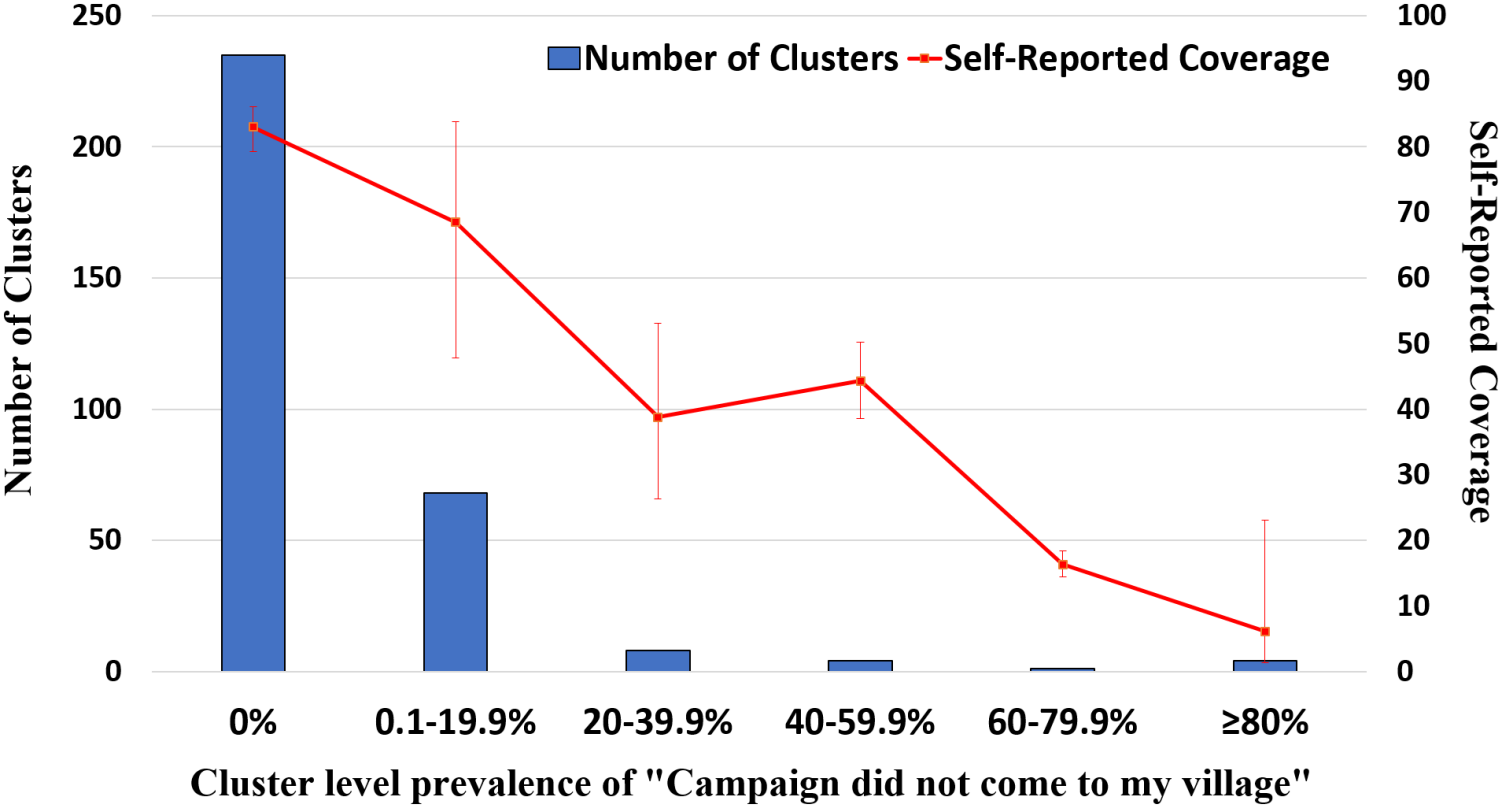
Cluster-level distribution of self-report of “campaign did not come to village,” Amhara, Ethiopia, 2016.

Among individuals who reported not being offered the drug, 20.6% (95%CI: 11.5–34.1%) of respondents said the campaign did not come to their village. The majority of selected survey clusters 235/320 (73.4%) had no one report that the campaign did not come to the village (Fig 4). Among clusters with a report of the campaign not coming to the village, 5.3% (17/320) of clusters had greater than 20% of the individuals surveyed reporting this as the reason they were not offered drug. Furthermore, there were 4 villages where greater than 80% of surveyed individuals gave this response. Two of these 4 clusters were located in two different districts of North Gondar zone, and two were located within the same district of West Gojjam zone.

## Discussion

Coverage surveys provide trachoma programs with a tool to estimate MDA coverage, to validate routine reports of administrative coverage, and to better understand barriers to high coverage. Following the 2016 trachoma MDA campaign, the self-reported MDA coverage in Amhara was greater than 80% in five of the ten zones and 76.8% region-wide. Zonal administrative coverage reports, however, were all greater than 90% and were consistently higher than coverage by self-report. Programmatic efforts should be made to better understand the reasons for these disparities. Barriers to higher coverage were most often provider-related, and therefore may be more amenable to programmatic improvements such as increased information and accessibility for all beneficiaries.

This coverage survey was conducted as part of an ongoing monitoring strategy within the Amhara Trachoma Control Program. The Carter Center provided the Amhara Regional Health Bureau with support to implement MDA, and separately, also funded, designed and executed this survey in collaboration with the Regional Health Bureau. The Carter Center has monitored MDA coverage in Amhara through various surveys since early in the program. In one random-cluster survey conducted in 2011 in East Amhara, district-level self-reported coverage in five districts ranged from 80% to 94.4% [14]. The following year, a second survey, using a 30x7 sampling technique in five different districts in West Amhara, found coverage estimates between 79.5% and 92.2% [14]. In the current survey, 5 of 10 zones had a coverage of less than 80%, and four of these were in West Amhara, which received its MDA in January instead of November for the first time in 8 years. Although this may have contributed to the lower coverage in that sub-region, programmatic improvements are still needed region-wide. In trachoma hyperendemic areas such as Amhara, however, it is still unclear whether an annual MDA strategy can achieve elimination as a public health problem, even with high coverage [15–18]. Further enhancements in antibiotic, facial cleanliness and environmental improvement interventions may be warranted.

Reported administrative drug coverage was consistently higher than the population based self-reported coverage estimates obtained by this survey. Discrepancies between administrative and self-reported coverage from within trachoma programs have been reported previously in Nigeria and South Sudan as well as previously in Amhara [6,9,14]. Possible explanations for the observed discrepancies in Amhara could include a miscalculation of the number of doses distributed, out-of-date or inaccurate census records, and incorrect adjustment of census data to account for yearly population changes in an area [7]. Manipulation of either doses distributed or adjusted populations could also have occurred, either at the distribution sites or at the district or zonal level offices. Although measuring coverage through either self-report or through administrative data has its limitations, trachoma programs can take clearly defined steps to better understand their administrative data systems. Further auditing of the drug distribution process either through registration book reviews or through data reporting assessments should help to improve the quality of administrative coverage data so that trachoma program managers can more effectively monitor the quality of their MDA campaigns [19].

Measuring coverage via heads of household self-report, or through including data from proxy response, resulted in similar estimates to those from self-report of the entire survey population. This may have been expected as randomized trials have demonstrated that MDA participation clusters within households [20]. These results also suggest that the extra effort required to collect proxy responses had little effect on drug coverage estimates for each zone. Further research comparing proxy responses to recorded medication registration book data would help in understanding both the accuracy of proxy responses and the utility of including such questions in coverage surveys. Ultimately, all three ways of measuring self-reported drug coverage in Amhara arrived at similar conclusions indicating good internal consistency of survey questions.

The identified barriers to reaching beneficiaries during the MDA campaign were often factors amenable to programmatic improvements. Being away from a village during drug distribution is a common problem for MDA campaigns and, if travelers are mingling with other untreated individuals, they could be a source of reinfection for the community once they return [21–25]. Lack of information about drug distribution is another important concern for MDA campaigns including those for trachoma [6,9,10,23,26,27]. Planning for effective social mobilization techniques to ensure individuals have adequate, advanced information about the time and location of the MDA campaign should be emphasized during future MDA campaign training. Zonal and district health offices should also work with HEWs to design local strategies to reach those who are not easily able to reach the distribution site, including individuals who may be old, sick or marginalized within their communities. Only a small proportion of individuals reported refusing MDA during the 2016 campaign. Education messages targeting side effects and strengthening alternative drug distribution options such as TEO for individuals who are sick, could enhance drug coverage in future MDAs.

Nearly 21% of individuals who reported that they were not offered MDA drugs said it was because the campaign did not come to their village. In some surveyed clusters only a few individuals reported this as a reason for not being offered drug, which contrasted with most other responses in the cluster. These individuals could possibly be considered refusers, yet ones who do not openly report refusing, possibly due to desirability bias. Another possibility is that some households may be removed, either spatially or socially, from the main village, and thus may have felt the campaign had not reached them. A few of the selected clusters had a high proportion of individuals who responded with this response, suggesting that the campaign may have truly missed their village. These villages could represent a source of reinfection in a district after an MDA. Future coverage surveys could be better designed to allow for the quick relay of this type of information to local health officials to allow for possible “mop-up” distributions while MDA campaigns are still ongoing [28].

Coverage surveys have several limitations including recall and desirability bias. This survey attempted to ameliorate the effects of recall bias by conducting the survey within three weeks after the MDA campaign and by presenting the pink azithromycin pill to the respondents to aid recall. The trachoma MDA campaign in Amhara is not currently integrated with MDA campaigns for other diseases, making confusion about specific medications unlikely. Furthermore, a recent study has demonstrated over 95% concordance between treatment registers and self-reported recall at one month after an MDA campaign for NTD medications suggesting recall may be fairly accurate [29]. This survey attempted to reduce desirability bias by using survey teams who were not part of the MDA campaign and by not allowing HEWs or other campaign officials into survey households. Survey teams did not give medications to those who reported not having participated in the campaign to discourage underreporting. Individuals or households who were not present during the survey may have been more likely to have been those which who not present during the MDA. To attempt to reduce this bias, within surveyed segments, if a household was empty during the survey, it was not replaced with a household with occupants present.

The results of this survey, which used a population-based probability sample representing all ten zones in Amhara, will allow zonal health officers to directly improve the implementation of future trachoma MDA campaigns, particularly in those zones with the lowest coverage. These results may be useful for other regions of Ethiopia which are currently scaling up their trachoma MDA programs.

## Supporting information

S1 Table Reasons for not attending trachoma MDA by zone in a) West Amhara sub-region, and b) East Amhara sub-region, Amhara, Ethiopia, 2016. (DOCX)
