# Supplementary material for: Population-based coverage survey results following the mass drug administration of azithromycin for the treatment of trachoma in Amhara, Ethiopia
Source: PLoS Negl Trop Dis. 2018 Feb 16;12(2):e0006270. doi: 10.1371/journal.pntd.0006270 (PMC5833287; doi:10.1371/journal.pntd.0006270)
Supplement: S1 Table — (DOCX) [file pntd.0006270.s001.docx]

Supplemental Table 1. Reasons for not Attending Trachoma MDA by Zone in a) West Amhara Sub-region, and b) East Amhara Sub-region, Amhara, Ethiopia, 2016

| a) | Awi | East Gojjam | North Gondar | South Gondar | West Gojjam |
| --- | --- | --- | --- | --- | --- |
| Reasons for not attending MDA | Percent  (95%CI) † | Percent  (95%CI) † | Percent  (95%CI) † | Percent  (95%CI) † | Percent  (95%CI) † |
| Physically unable to get to the distribution site | 14.5% (5.5-33.1%) | 7.0% (2.5-18.1%) | 8.2% (4.7-14.1%) | 24.2% (16.9-33.3%) | 30.3% (11.3-59.7%) |
| I did not know about the MDA campaign | 12.9% (6.1-25.2%) | 35.7% (17.1-60.0%) | 13.9% (4.0-38.6%) | 18.3% (12.7-25.6%) | 19.3% (11.3-31.0%) |
| Traveling during the MDA | 39.0% (17.9-65.3%) | 13.5% (4.9-32.2%) | 44.8% (30.9-59.5%) | 13.9% (8.8-21.3%) | 17.5% (7.6-35.6%) |
| I did not want the medication | 11.2% (5.2-22.6%) | 20.9% (15.5-27.6%) | 18.9% (14.7-23.9%) | 8.4% (4.1-16.4%) | 7.4% (3.1-16.6%) |
| Chores or duties for the household | 9.0% (4.4-17.4%) | 10.0% (5.1-18.8%) | 12.1% (7.6-18.9%) | 14.1% (8.8-22.0%) | 12.2% (7.2-20.0%) |
| Attended social or religious events elsewhere | 13.5% (8.4-21.0%) | 12.9% (3.9-35.0%) | 2.2% (0.8-5.7%) | 21.1% (13.1-32.1%) | 13.3% (6.4-25.8%) |
| Other* | -- | -- | -- | -- | -- |

† Weighted zonal estimate. Multilevel survey design accounted for in analysis

| b) | North Shoa | North Wollo | Oromia | South Wollo | Waghimra |
| --- | --- | --- | --- | --- | --- |
| Reasons for not attending MDA | Percent  (95%CI) † | Percent  (95%CI) † | Percent  (95%CI) † | Percent  (95%CI) † | Percent  (95%CI) † |
| Physically unable to get to the distribution site | 6.5% (3.8-10.7%) | 35.0% (22.6-49.8%) | 14.4% (7.5-26.1%) | 19.4% (14.5-25.4%) | 11.2% (5.0-23.5%) |
| I did not know about the MDA campaign | 16.8% (6.7-36.0%) | 25.6% (14.0-42.2%) | 16.9% (8.9-29.8%) | 16.4% (5.2-41.1%) | 6.1% (2.3-15.3%) |
| Traveling during the MDA | 18.9% (12.3-27.9%) | 17.1% (7.1-35.7%) | 32.7% (20.8-47.3%) | 22.3% (17.6-27.9%) | 37.6% (24.4-52.9%) |
| I did not want the medication | 38.3% (31.1-45.9%) | 10.6% (2.5-35.6%) | 12.6% (7.7-20.0%) | 9.6% (5.2-17.1%) | 11.8% (5.5-23.5) |
| Chores or duties for the household | 14.1% (7.8-24.2%) | 6.8% (5.3-8.8%) | 16.9% (7.2-35.0%) | 17.1% (11.1-25.5%) | 23.3% (9.9-45.6%) |
| Attended social or religious events elsewhere | 2.2% (0.5-9.0%) | 3.9% (2.2-6.9%) | 2.0% (0.3-14.7%) | 7.6% (4.2-13.2%) | 6.2% (3.3%-11.2%) |
| Other* | 3.3% (1.1-9.4%) | 1.0% (0.3-3.8%) | 4.4% (2.5-7.7%) | 7.7% (2.3-22.7%) | 3.9% (1.1-12.6%) |

† Weighted zonal estimate. Multilevel survey design accounted for in analysis. *Other response not available in West Amhara.
